# Assessing the interlinkage of green and blue water in an arid catchment in Northwest China

**DOI:** 10.1007/s10653-019-00406-3

**Published:** 2019-09-07

**Authors:** Ganquan Mao, Junguo Liu, Feng Han, Ying Meng, Yong Tian, Yi Zheng, Chunmiao Zheng

**Affiliations:** 1 School of Environmental Science and Engineering, Southern University of Science and Technology, 1088 Xueyuan Avenue, Shenzhen, 518055 China; 2 School of Water Resources and Hydropower Engineering, Wuhan University, Wuhan, 430072 China

**Keywords:** Ecosystem, Irrigation, Risk, Water competition, Water management

## Abstract

Water resource assessment is crucial for human well-being and ecosystem health. Assessments considering both blue and green water are of great significance, as green water plays a critical but often ignored role in the terrestrial ecosystem, especially in arid and semi-arid regions. Many approaches have been developed for green and blue water valuation; however, few approaches consider the interrelationship between green and blue water. This study proposed a new framework for green and blue water assessment by considering the interactions between green and blue water and the connections between human and natural ecosystems in an arid endorheic river basin where hydrological cycling is dramatically altered by human activities. The results show that even though green water is the dominant water resource, blue water is also critical. Most of the blue water is redirected back into the soil through physical and human-induced processes to meet the water demand of the ecosystem. The blue and green water regimes are found to be totally different in different ecosystems due to the temporal and spatial variability in water supply and consumption. We also found that humans are using an increasing proportion of water, resulting in decreasing water availability. Extensive water use by humans reduces the water availability for the natural ecosystem. Approximately 38.6% of the vegetation-covered area, which is dominated by farmland and forest, may face a moderate or high risk of increased conflict and tension over freshwater. This study provides crucial information to better understand the interactions between green and blue water and the relations between humans and nature by explicitly assessing water resources. It also provides crucial information for water management strategies that aim to balance humankind and nature.

## Introduction

Renewable freshwater is a foundation for both terrestrial and aquatic ecosystems (Jackson et al. 2001). A sufficient water supply is essential for the survival of all organisms, including humans (Lu et al. 2019; Oki and Kanae 2006). Today, freshwater is becoming increasingly scarce in many regions of the world. Water scarcity has become a worldwide problem, and its intensity has increased due to climate change and human activities, e.g. unsustainable land and water management (Liu et al. 2017; Veldkamp et al. 2017). This has become a major constraint to our socio-economic development. As the demand for water increases due to economic development and population growth, the competition for water resources between agriculture, livestock, fisheries, forestry, energy and other sectors also increases, with unpredictable impacts on livelihoods and environments (Guo et al. 2019; Jiang et al. 2019; Johansson et al. 2016; Ye et al. 2019). Therefore, renewable freshwater availability assessments are crucial for better water resource management and for resilience against water scarcity under the changing environment (Rockström et al. 2009a).

Freshwater essentially stems from precipitation. Conceptually, fresh water resources can be divided into green water resources and blue water resources. Green water is site-specific precipitation that does not run off but more or less temporarily contributes to soil water storage and is eventually consumed by ecosystems through evapotranspiration, while blue water is surface and groundwater that is stored in rivers, lakes, aquifers and dams and can be extracted for human use (Falkenmark and Rockström 2006). Green water consists of two components: productive green water, i.e. transpiration from biomass production in terrestrial ecosystems, and the non-productive green water, i.e. interception and soil evaporation (Rockström and Falkenmark 2000). Early conventional water resource assessment and management considered only blue water, while green water was ignored (Eastham et al. 2008; Shiklomanov 2000; Xu and Singh 2004). However, green water plays a critical role in terrestrial ecosystems, especially in arid and semi-arid regions (Liu et al. 2009a; Rockström et al. 2007; Rost et al. 2008; Schyns et al. 2015). Water resource assessments considering both blue and green water have become increasingly diversified after the concept of green water was introduced by Falkenmark (1995). In particular, after Falkenmark and Rockström (2006) conceptualized an expanded green and blue water assessment approach for water resource planning and management, a great number of novel green and blue water studies have been performed. For instance, Schuol et al. (2008) combined the soil and water assessment tool (SWAT) model and a geographic information system (GIS) interface with the SUFI-2 calibration procedure and successfully simulated monthly blue and green water resources for the entire African continent at a detailed sub-basin level. Liu et al. (2009a, b) used a GIS-based erosion-productivity impact calculator (GEPIC) model to calculate the global consumption of green and blue water for crop production and highlighted the importance of green water, especially for rain-fed agriculture. Zang et al. (2012) simulated the spatial and temporal distribution of blue and green water for an inland river basin in Northwest China; the spatial variation and temporal trend of green water, blue water and the green water coefficient were explicitly discussed. Lathuillière et al. (2016) investigated the trade-offs between blue water resources and green water resources in the Amazon basin in the light of future agricultural production and potential irrigation to assess costs and benefits to terrestrial ecosystems, specifically regarding land and biodiversity protection and regional precipitation recycling. Weiskel et al. (2014) used a distributed landscape water balance model to simulate green and blue water fluxes across the USA and developed a classification of hydroclimatic regimes based on simulated green and blue water fluxes. In addition, many other studies on green and blue water resources have been performed (Chukalla et al. 2015; Johansson et al. 2016; Mekonnen and Hoekstra 2011; Rost et al. 2008; Schyns et al. 2015; Sulser et al. 2010). These green and blue water-related studies can generally be categorized into two groups depending on the different perspectives of the investigations: (1) assessments of green and blue water availability and dynamics with a hydrological model or water balance model; and (2) assessments of green and blue water use or consumption with a water resource model, agricultural model or dynamic vegetation model. Both strategies are important to elucidate scientific information for water management, as the water resources from precipitation are recycled exclusively through water consumption by evapotranspiration processes (Lathuillière et al. 2016). In addition, many studies have been conducted to investigate the climate and anthropogenic impacts on green and blue water and the corresponding environmental impacts (Chen et al. 2014; Liu 2009; Liu et al. 2009b; Quinteiro et al. 2018; Zang et al. 2015). However, few studies have focused on the interconnection between green water and blue water and the connection between humans and nature.

Green and blue water are interlinked; changes in blue water also drive changes in green water and vice versa (Rockström et al. 2009b). Several studies have shown that human-driven changes in land cover, land use and water use alter the evaporation flux from the land to atmosphere (Destouni et al. 2012; Gordon et al. 2005; Jaramillo et al. 2013; Wang and Hejazi 2011) as well as the amount of moisture stored in the unsaturated soil layer, as the soil moisture feeds the evaporation flux (Destouni and Verrot 2014). Such green water-related evaporation changes also affect run-off generation and therefore the consumptive use of blue water (Destouni et al. 2012; Jaramillo et al. 2013; Jaramillo and Destouni 2014). Thus, it is necessary to shift the strategy of traditional green and blue water resource assessments by also considering the interactions between them, i.e. beyond the water balance (McDonnell 2017).

On the other hand, we are now in an era in which more data have become available for hydrological simulation at different scales (basin, continental, or even global scales) (Lee et al. 2004). Although green and blue water availability can be sufficiently captured by the water balance accounting model, which requires the smallest datasets, compartmentalization of the parts of the terrestrial water cycle could have a controlling influence on green and blue water storage dynamics in time and space (Cravotta et al. 2014). Various types of data allow us to construct sophisticated models that depict the hydrological processes in an explicit way by tracking all the necessary water fluxes and storage processes in hydrological cycling. Thus, understanding the interconnection between green water and blue water and the relation between humans and nature is necessary even it is more challenging than only the bulk quantification of water availability or water consumption in water resources assessments.

In this study, we assessed green and blue water by considering the interactions between them and emphasizing the relation between humans and nature in an arid endorheic river basin in China that is suffering from strong anthropogenic impacts. In arid and semi-arid areas, water resources are critical, and both human and natural ecosystems are vital to the sustainability of local societies and ecosystems. Human activities further alter green and blue water interactions, imposing an additional stress on the water-limited environment. Understanding the complicated relationships between green and blue water and the connection between humans and natural ecosystems is of great importance to water resource management and ecological conservation in such areas.

This study aims to consider the interactions between green water and blue water in the water cycle and apply an integrated surface water and groundwater model for water resource assessment with two main objectives: (1) determine how well integrated surface water and groundwater hydrological modelling efficiently and effectively simulates green and blue water dynamics while emphasizing the interlinkages between them in an arid endorheic river basin by explicitly considering irrigation, water diversion and groundwater pumping; and (2) determine how such green and blue water assessments could support basin-scale water resources management to address human-nature water conflicts in an arid endorheic catchment.

## Data and methods

### Study area

The Heihe River Basin (HRB) was selected as the study area for this work for the following reasons: (1) the HRB is located in the arid area, and water in this area is limited; (2) the HRB is impacted heavily by human activities, and hydrological cycling is dramatically altered (Zhou et al. 2015b); and (3) the HRB has strong groundwater and surface water exchanges that influence the interactions between green water and blue water (Zhu et al. 2008).

**Fig. 1:**
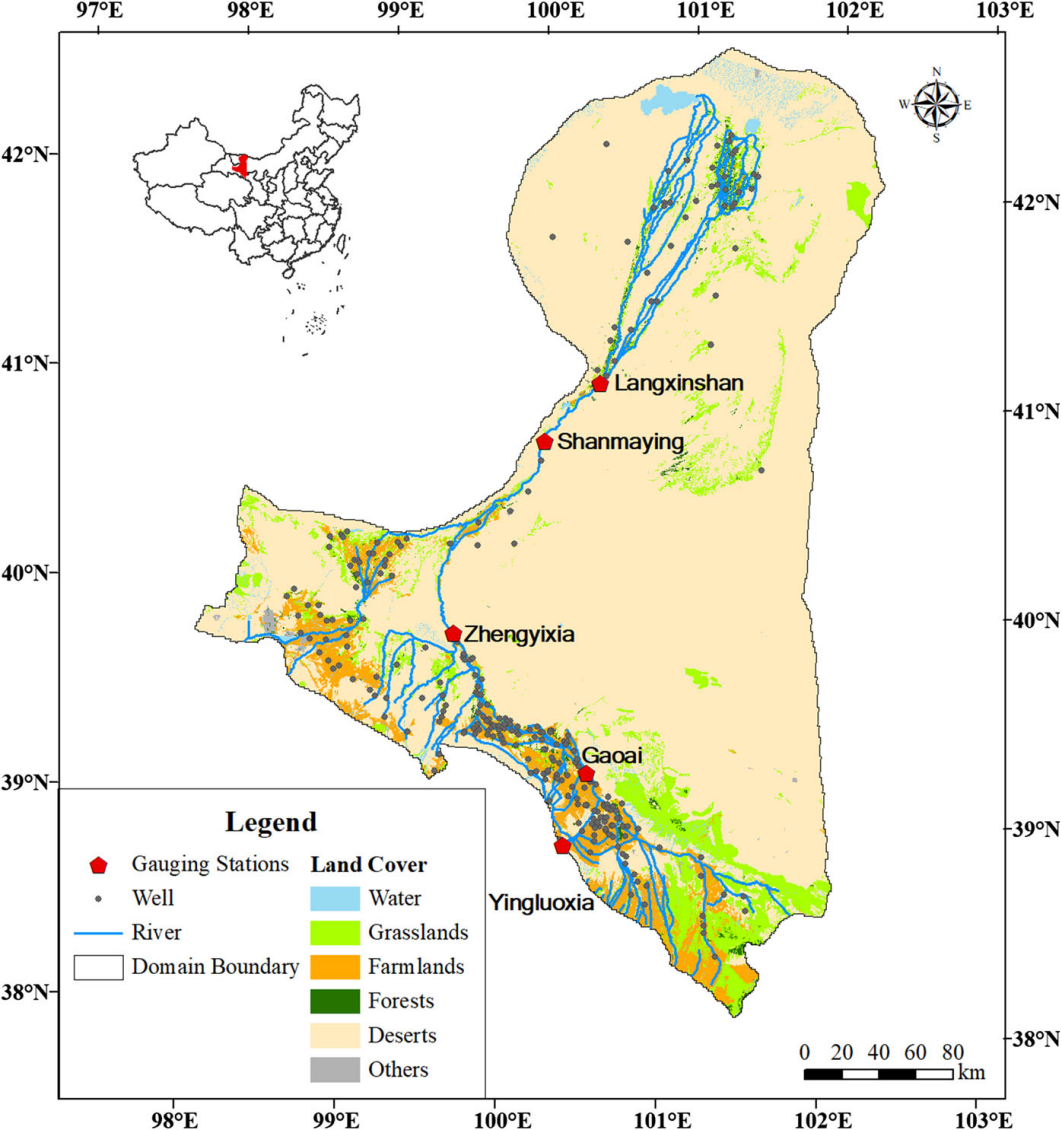
The study domain

The HRB is the second largest endorheic river basin in China, with the majority located in Northwest China and a minor part in Mongolia. The Heihe River originates in the Qilian Mountains and discharges into Juyanhai Lake, with a total basin area of 0.24 million km^2^. Our research domain covers the middle and lower HRB plus a portion of the Badain Jaran Desert, which is hydraulically connected with the HRB through its groundwater system (Fig. 1). The upper HRB was excluded from the domain because the upper HRB is still natural without human influences and has sufficient water resources for the ecosystem. The total area of the domain is 90,589 km^2^. The domain consists of different types of land use/land cover (LULC), such as farmland (5.6%), grassland (5.9%), forest (1.1%), water bodies (1%), desert (82.1%, including the Gobi Desert) and others (4.2%). The farmland, which is used mainly for corn and winter-wheat planting, is intensively irrigated. Irrigation also happens in the grassland and forest areas in the middle and lower HRB for two reasons. First, the Chinese government launched a water use policy in the late 1990s to protect the environment in the middle and lower HRB for the conservation of *Populus euphratica*, a typical plant in the HRB (Sun et al. 2016; Zhang et al. 2015). Thus, a portion of the water in the HRB is used to irrigate the grasslands and forests (Nian et al. 2014). Second, there are also grasslands and forests located in the irrigation districts in the middle HRB (Li et al. 2018) that receive the irrigated water from the irrigation system.

The climate and hydrological conditions and the LULC are slightly different for different parts of the domain. For instance, the middle HRB contains a large area of alluvial fans and floodplains and is nearly completely composed of intensively irrigated farmland. The elevation of the middle HRB ranges from 1200 to 3880 m. The annual average temperature is approximately 8 °C, while the annual precipitation varies in space from 50 to 400 mm, with an average of 145 mm. The lower HRB is a vast Gobi Desert area that is relatively flat with a mean elevation of approximately 1000 m. The vegetation mainly develops in the floodplains along the main river. The annual average temperature is approximately 10 °C, and the annual precipitation is extremely low. In some regions, the annual average precipitation is less than 50 mm (Wang and Niu 2016; Zang and Liu 2013). Another large part of the domain is the Badain Jaran Desert, which has many tall stationary dunes and numerous scattered lakes (probably fed by groundwater) (Jiao et al. 2015). The annual average temperature is approximately 9 °C, and the annual precipitation is approximately 110 mm. Since we were interested in only a part of the entire HRB, our study area was not physically contained. There are more than 30 perennial rivers, including the main Heihe River, that bring approximately 3.5 billion cubic metres per year (m^3^/year) of surface run-off from the Qilian Mountains (upper HRB) to the research domain.

### Model

A sophisticated distributed hydrological model was selected for this research for two reasons. (1) This study aimed to assess water resources by investigating the interlinkages between green water and blue water. The selected model is capable of simulating all the necessary hydrological elements for this analysis due to the capacity for a detailed depiction of interactions between groundwater and surface water. (2) Gridded hydrological simulations from distributed models are preferred for spatial investigations of green and blue water because they reveal spatial heterogeneity.

An improved coupled groundwater and surface water flow (GSFLOW) model, which is capable of explicitly considering irrigation, water diversion and groundwater pumping, was used in this study (Tian et al. 2015a). The GSFLOW model integrates a hydrological Precipitation-Runoff Modelling System (PRMS) and a modular groundwater flow (MODFLOW) model, which produce a 2-D surface hydrology simulation and a 3-D groundwater simulation, respectively (Markstrom et al. 2008). In the surface domain, hydrologic response units (HRUs) are the basic computing units, which can be either regular grids or irregular polygons, while the subsurface domain is discretized into finite difference grids. To simulate the interactions between surface water and groundwater, a vadose zone between the soil zone and aquifer is defined in GSFLOW, which is handled by the unsaturated zone flow (UZF1) package (Niswonger et al. 2006) associated with MODFLOW. A “gravity reservoir” in the vadose zone is specified for each HRU in which the HRU exchanges water with the MODFLOW grid(s). The streamflow routing (SFR2) package (Niswonger and Prudic 2005) and lake (LAK3) package (Merritt and Konikow 2000) are integrated in GSFLOW to simulate streams and lakes, respectively. In reaches where the stream water is connected with the groundwater, the stream-aquifer exchange is calculated based on the head difference using Darcy’s law (Darcy 1856; Hubbert 1957). More details about GSFLOW and its improvements can be found in Tian et al. (2015b).

### Data sources

The improved GSFLOW model has a rich description of ecological and hydrological processes, and multisource data are desired for precise and thorough simulation. In this study, the data used for modelling were obtained from the Heihe Program Data Management Center (https://www.heihedata.org); the details are listed in Table 1. The data used in this study are grouped into two categories. The first category is the data for the model set-up and initial parameterization, which includes the digital elevation model (DEM) (Farr and Kobrick 2000) and land use (Hu et al. 2015a, b; Zhong et al. 2015), soil type (Dai et al. 2013; Wei et al. 2013), irrigation system (Hu et al. 2008), river network data and so on. These data are essential for the model to define the topography, drainage systems, boundary conditions, vertical segmentation of the subsurface and so on. The second category of the data is dynamic model inputs that consist of model-derived climate forcing (Xiong and Yan 2013), meteorological observation, surface water diversion and groundwater pumping information.

**Table 1:** Data sources for hydrological simulation

Category	Data	Time of data	Spatial resolution
Model set-ups	DEM	2000	90 m × 90 m
	Land use	2000, 2007, 2011	1:100,000
	Soil texture	2012	1 km × 1 km
	Normalized difference vegetation Index	2000–2012 (every 10 days)	1 km × 1 km
	River network	2000	1:100,000
	Irrigation system	2006	1:100,000
	Hydrogeology map	2002	1:500,000
	Borehole data	Multiple time spots	257 locations
	Boundary river inflow	2000–2012 (daily/monthly)	15 stations
	Boundary groundwater inflow	2000–2012 (yearly)	Boundary grids
Model inputs	Model-derived climate data	2000–2012 (six hours)	3 km × 3 km
	Meteorological observations	2000–2012 (daily)	19 stations
	Surface water diversion	2000–2012 (monthly)	46 districts
	Groundwater pumping	2000–2012 (yearly)	46 districts

### Framework of the green and blue water assessment

The improved GSFLOW model is applied and is driven by multisource data to simulate necessary water fluxes and water storage processes for the assessment. First, for the application of hydrological simulation for the entire domain, uniform 1 km × 1 km grids were used for both the surface and subsurface domains in the model. The entire research domain contains 90,589 pixels, and each grid cell in the surface domain represents an individual HRU. The flow paths across the HRUs are defined by using the Cascade Routing Tool (CRT) developed by Henson et al. (2013). The model has already been well calibrated and validated in this region by Tian et al. (2015a, b). In this study, the same set-ups and configurations, including the calibrated parameters, were applied to the model. Therefore, the model is suitable for the study area without any further parameter tuning. The model was run at a daily scale from 1 January 2000 to 31 December 2012, while the first year (2000) was treated as a “spin-up” period to eliminate the impact of initial conditions (mainly soil moisture) on the model simulations.

**Fig. 2:**
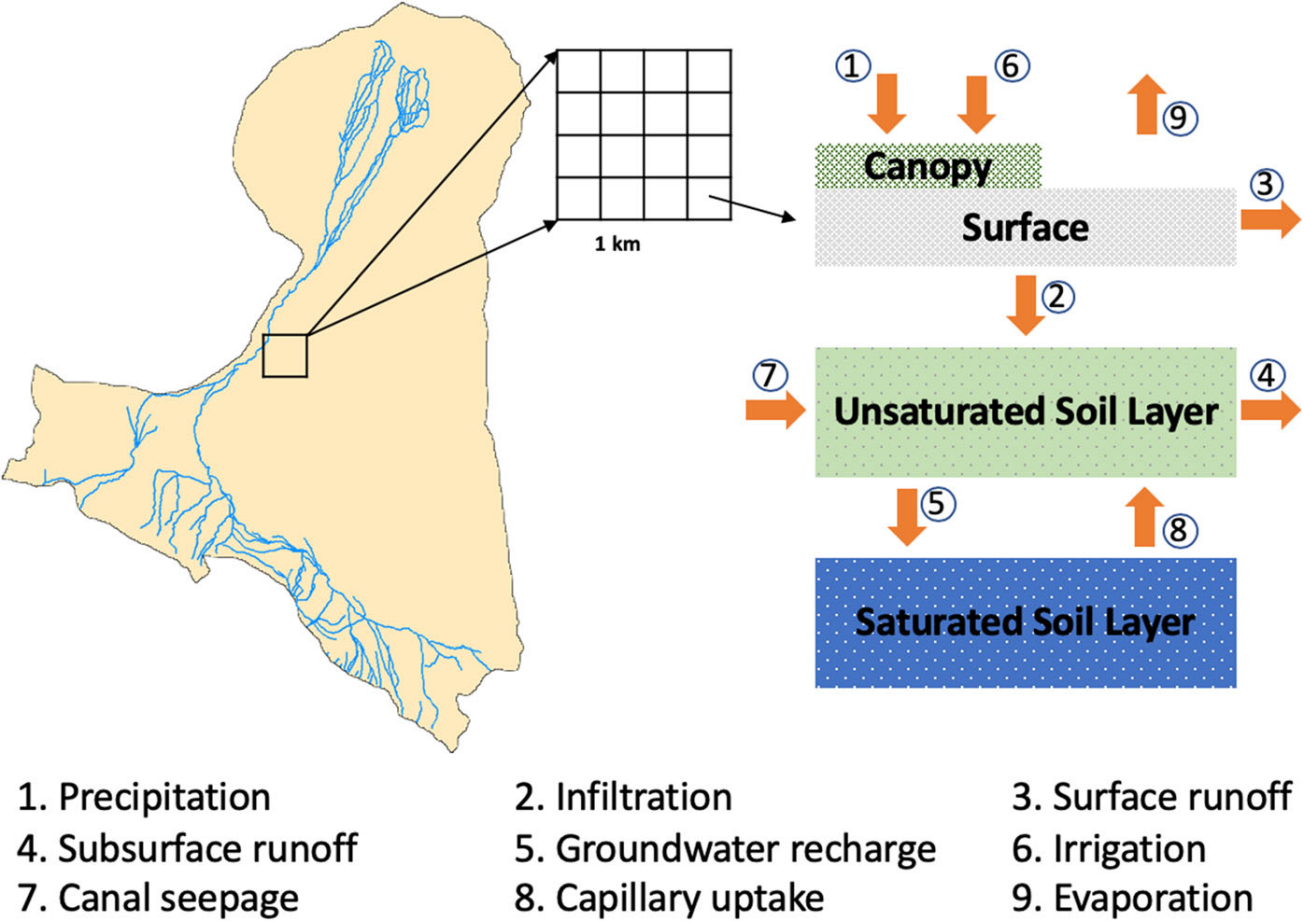
The framework for hydrological simulation in the research domain

Figure 2 shows the framework for the hydrological simulation of necessary variables. After the simulation, green and blue water resources are calculated for assessments followed the definition of Falkenmark and Rockström (2006). The complete strategy consists of the following steps that cover both water availability and water consumption:

1. Green water resources are calculated by summing the interception and soil moisture recharge simulated by the model for a certain period (e.g. an annual scale). It is important to state here that in the model, green water resources refer to only the water from precipitation that stored in the soil. The water that runs through the soil to recharge the groundwater or that is routed into the channel is excluded.
2. Blue water resources are calculated by summing the model-simulated surface run-off, subsurface run-off and groundwater recharge.
3. Water consumption is calculated by summing the evaporation, while green water consumption refers to the actual evaporation from green water resources, and blue water consumption refers to the actual evaporation from blue water resources.
4. Green and blue water resources and water consumption are first calculated for each pixel and then summed for analyses at different scales, e.g. the entire domain or different ecosystems.

### Green water coefficient and Blue Water Demand Index

Precipitation is separated into run-off and infiltration that recharges the soil when it reaches the surface (Rockström et al. 1999, b), which forms blue and green water. The green water coefficient (GWC) is defined as the ratio of green water resources to the total water resources (green water + blue water) from precipitation and can be calculated simply by the following equation (Liu et al. 2009a):

$${\text{GWC}} = \frac{{G_{{\text{p}}} }}{{B_{{\text{p}}} + G_{{\text{p}}} }}$$

where $B_{{\text{p}}}$ and $G_{{\text{p}}}$ are blue and green water resources from precipitation, respectively. GWC reflects the precipitation partitioning ratio and is often used to analyse green and blue water from the perspective of water availability or water supply (Chen et al. 2014; Liu et al. 2009a; Liu and Yang 2010; Zang et al. 2012). To assess the water resources from the perspective of water consumption, we defined a new index, called the blue water consumption ratio (BWCR). The equation of BWCR is described as follows:

$${\text{BWCR}} = \frac{{B_{{\text{c}}} }}{{B_{{\text{c}}} + G_{{\text{c}}} }}$$

where $B_{{\text{c}}}$ and $G_{{\text{c}}}$ are blue and green water consumption, respectively. BWCR reflects the dependence of water consumption on the blue water resources. A high BWCR value for a certain region indicates a high reliance on blue water in this region. In this study, the blue water consumption consists of human-induced blue water use, e.g. irrigation and canal seepage, and physically induced blue water consumption, e.g. capillary rise.

In this study, thresholds of 0.5 and 0.75 are used to classify the risk levels. A BWCR equal or greater than 0.75 indicates an area with a very high potential risk of increased competition and conflict over water resources. A BWCR between 0.75 and 0.5 implies an area with moderate potential risk, and a BWCR less than 0.5 represents an area with low risk, as these areas rely mainly on green water rather than blue water.

## Results and discussion

### Domain-averaged green and blue water flow chart

**Fig. 3:**
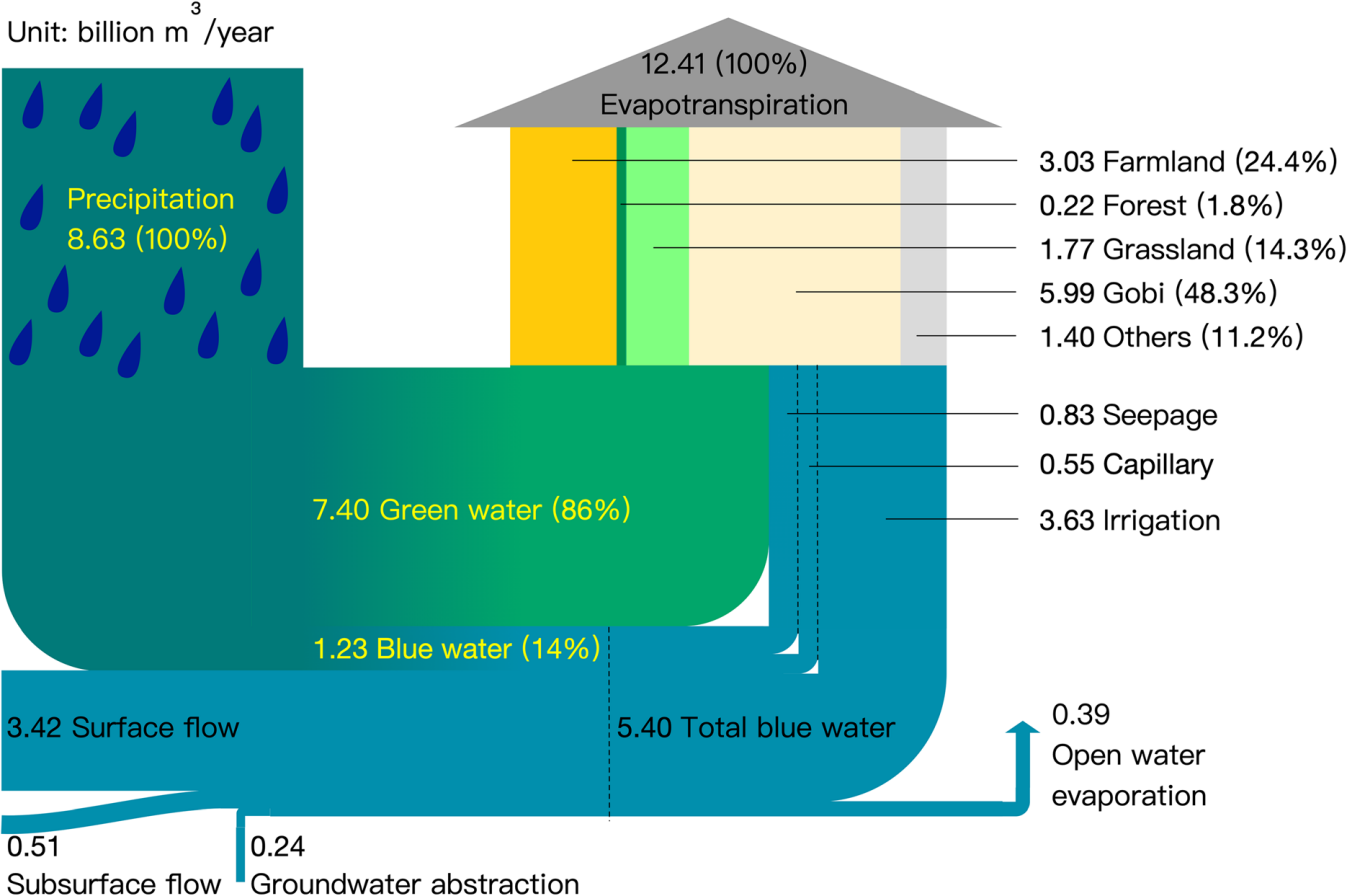
The green and blue water flow chart for the entire domain on an annual scale. The left side shows the water resource availability of the domain, which consists of water from precipitation, surface and subsurface flow from upstream as well as groundwater abstraction. The large vertical arrow on the right side shows the water consumption by the different ecosystems (land use), and the small vertical arrow shows the water consumption in the open water region. Blue colours indicate blue water flows, and green colours indicate green water flows. A mixture of blue and green implies precipitation, while a gradient transition from blue to green shows the transformation of blue water to green water. Numbers in parentheses indicate the percent of the total, the yellow text represents the water resources from precipitation, and the black text represents water consumption. The width of the band reflects the quantification of each element

To investigate the green and blue water resources in an explicit way, a detailed green and blue water availability and consumption analysis was performed, and the results are shown in the green and blue water flow chart (see Fig. 3). The results are derived from the hydrological simulations from 2001 to 2012 and are shown on an annual scale. The annual average precipitation in the research domain is 95.3 mm, which is 8.63 billion m^3^ in volume. It was found that 86% (7.40 billion m^3^/year) of the water from precipitation forms green water resources that are stored in soil, and only 14% (1.23 billion m^3^/year) of precipitation runs off when it reaches the surface. In addition to the water resources from precipitation, there is also a large amount of water (3.93 billion m^3^/year), accounting for 72.8% of the total blue water and 30.7% of the total water resources, from upstream of HRB that charges the research domain. At the global scale, approximately two-thirds of the water resources are stored as green water consumption (Gerten et al. 2005; Rost et al. 2008). In this arid river basin, the water resources are dominated by green water with an extremely high GWC of 0.86. Even considering the blue water replenishment from upstream, the green water proportion of the total water resources is still high, at 57.8%. This emphasizes the importance of green water resources, as they are the main water resources in this area. This finding is also consistent with other studies in this region (Zang et al. 2015; Zang and Liu 2013; Zuo et al. 2015).

Our research domain is an endorheic river basin; therefore, there is no water flow out of the area, and all the water entering this area will eventually evaporate into the atmosphere. A total of 0.39 billion m^3^/year evaporates directly from open water bodies into the atmosphere; however, this accounts for only 3% of the total water consumption. The rest of the water resources (97%) are consumed by terrestrial ecosystems and other land uses, e.g. bare soil and urban areas, in the region. This phenomenon is due to the limited open water area in arid or semi-arid regions (Sánchez-Carrillo et al. 2004). The average annual total water consumption by the land for the study area is 12.41 billion m^3^/year, and the consumption rates for each ecosystem, i.e. farmland, forest, grassland and desert, are 3.03 billion m^3^/year, 0.22 billion m^3^/year, 1.77 billion m^3^/year and 5.99 billion m^3^/year, respectively. The desert ecosystem we used hereafter refers to all the types of desert as there are different types of deserts in our study area. The desert ecosystem consumes the most water (48.3% of the total water consumption from the land) due to its large area, followed by the farmland ecosystem (24.4%), which is partly due to intensive irrigation that redirects the blue water resources to soil water storage (Ge et al. 2013). In addition, approximately 1.40 billion m^3^ (11.2%) of water is consumed each year by other land uses, including bare soil and urban areas. The total water consumption (12.80 billion m^3^/year) is higher than the total water availability (12.56 billion m^3^/year), as approximately 0.24 billion m^3^/year (2.7 mm in depth) of groundwater is abstracted each year. It is important to point out that the total water consumption from land (12.41 billion m^3^/year) is 67% greater than the green water storage (7.4 billion m^3^/year), implying that a large amount of additional blue water resources is needed for this region to meet the consumption by land ecosystems.

For the entire domain, there is 5.40 billion m^3^/year of blue water in total, while only 7.2% (0.39 billion m^3^/year) evaporates from open water bodies. The remainder of the water is redirected to the soil, which recharges the soil moisture and replenishes green water resources that are eventually consumed by land ecosystems. The redirection of blue water resources is mainly driven by three factors, i.e. irrigation, canal seepage and capillary uptake, due to human activities and hydrological processes. Approximately 3.63 billion m^3^ of blue water is used each year for irrigation through water pumping from wells (0.82 billion m^3^) and water diversion from rivers (2.81 billion m^3^). Due to the irrigation systems, 0.83 billion m^3^/year of blue water is additionally redirected to the soil through canal seepage. In addition, 0.55 billion m^3^ of blue water recharges the soil moisture through capillary rise. All these blue water resource redirections that replenish green water resources are critical for ecosystems, especially agriculture ecosystems, as the water demand is substantially higher than the water stored in the root zone layer (green water resources).

Our study area covered only the middle and lower HRB, although the water resources from precipitation are still the main water resources for the study area; additional water from upstream (the upper HRB) is also crucial for the ecosystems in this region (Gao et al. 2014), and the run-off generated upstream could have profound impacts on the ecosystems in the middle and lower HRB. The imbalance between water availability and water consumption causes an average groundwater depletion rate of 0.24 billion m^3^/year. This groundwater depletion had implications for water management in this region as it could cause serious problems for both humans and ecosystems in future. Furthermore, the inequality between green water storage and green water consumption implies an extensive redirection of blue water to recharge soil water moisture. The detailed water flow chart analysis helps us to further understand the eco-hydrological processes beyond the water balance and provides crucial information for water management, especially for identifying the interlinkages between green and blue water from the perspective of both water supply and water consumption.

### Analysis of spatial and temporal variability of the water resources

**Fig. 4:**
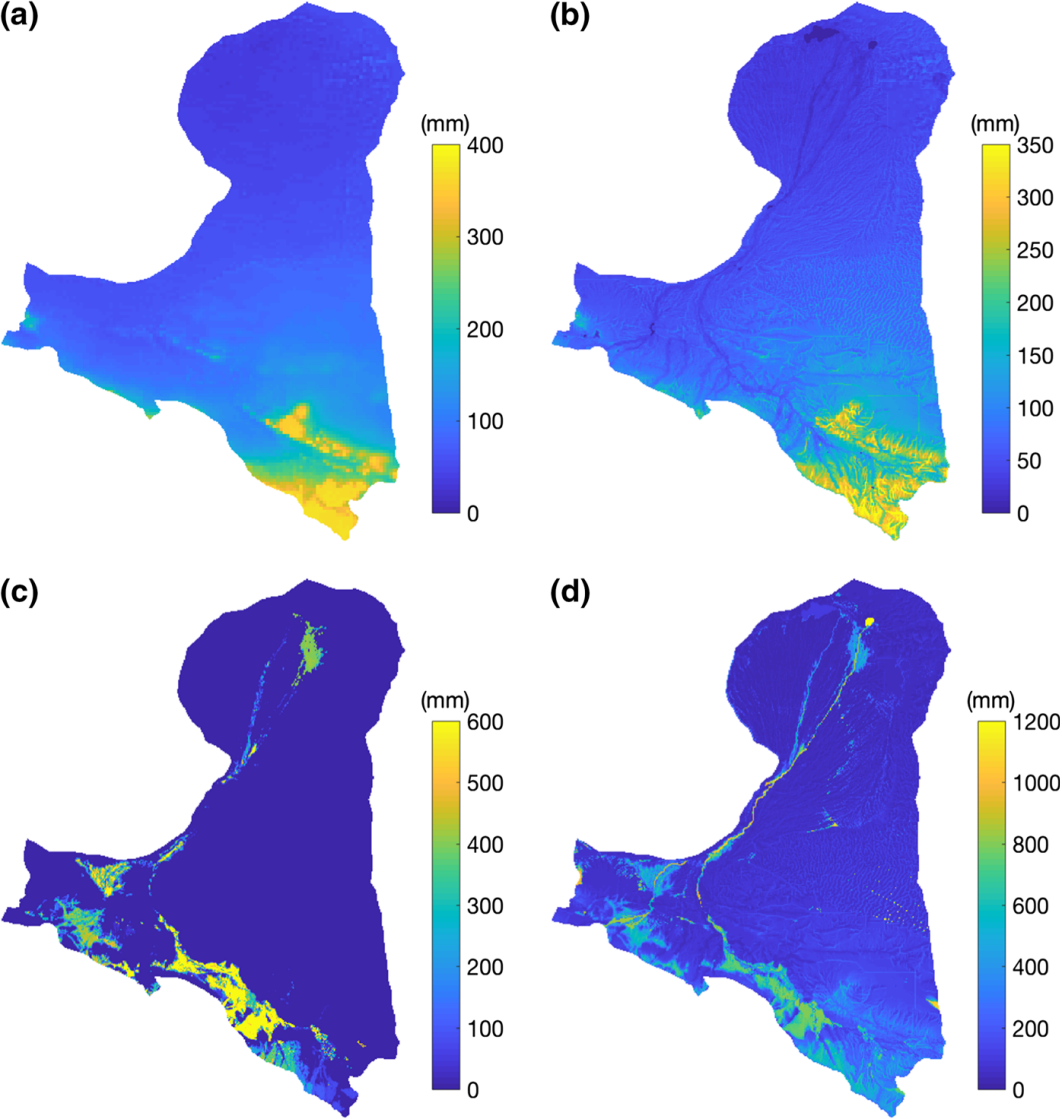
Spatial analysis of water supply and water consumption including **a** total water resources from precipitation, **b** green water resources from precipitation, **c** irrigation and **d** total water consumption in the research domain. The results are shown on an annual scale with the average mean based on the data from 2001 to 2010. The colour axis is scaled differently based on the different data ranges

In addition to assessing the water resources at the basin scale, it is also necessary to reveal their spatial and temporal patterns, which provide information in a more detailed way. First, the spatial patterns of critical water elements, including total water resources from precipitation, green water resources, irrigation and total water consumption, were investigated and are shown in Fig. 4.

All four of these variables are shown at an annual scale at a spatial resolution of 1 km, and all of them clearly vary in space. Figure 4a shows the averaged total water resources the entire domain can receive per year from precipitation. The water resources show extensive variability, and the south-east area of the domain receives more precipitation than the other areas. In the northern study area, the precipitation received is quite low. In some regions, the precipitation is even less than 50 mm/year. Figure 4b shows the green water that can be received for the area. The green water resources follow the exact pattern of precipitation because they come from precipitation. Additionally, a clear river network is shown in the map because the precipitation that directly reaches the channel is calculated as blue water instead of green water. The map of blue water from precipitation is not shown here, as the blue water can be simply calculated by subtracting the green water resources from precipitation from the total water resources from precipitation.

Figure 4a and b shows water resources, including total water resources and green water resources, from precipitation, while (c) shows the human-induced water allocation, i.e. irrigation, and (d) shows the total water consumption, i.e. evaporation. Figure 4c and d shows similar patterns since irrigation directly impacts evaporation. Additionally, differences caused by heavy precipitation in this region can be seen in the south-eastern part of the domain. By comparing the precipitation (Fig. 4a) and irrigation maps (Fig. 4c), the results show that irrigation is the dominant water resource for most of the irrigated area. In some regions, the magnitude of irrigation is even larger than that of precipitation. The differences between the precipitation map (Fig. 4) and evaporation map (Fig. 4 d), especially the large difference in the range, show the large imbalance between water resources received from precipitation and water consumption. This reflects the fact that the upstream inflow, the blue water from outside of the domain, is crucial for the region to meet water requirements. It also emphasizes the importance of redirecting blue water to recharging the soil moisture, which supports the ecosystem and bridges the spatial gaps between water supply and water use.

**Fig. 5:**
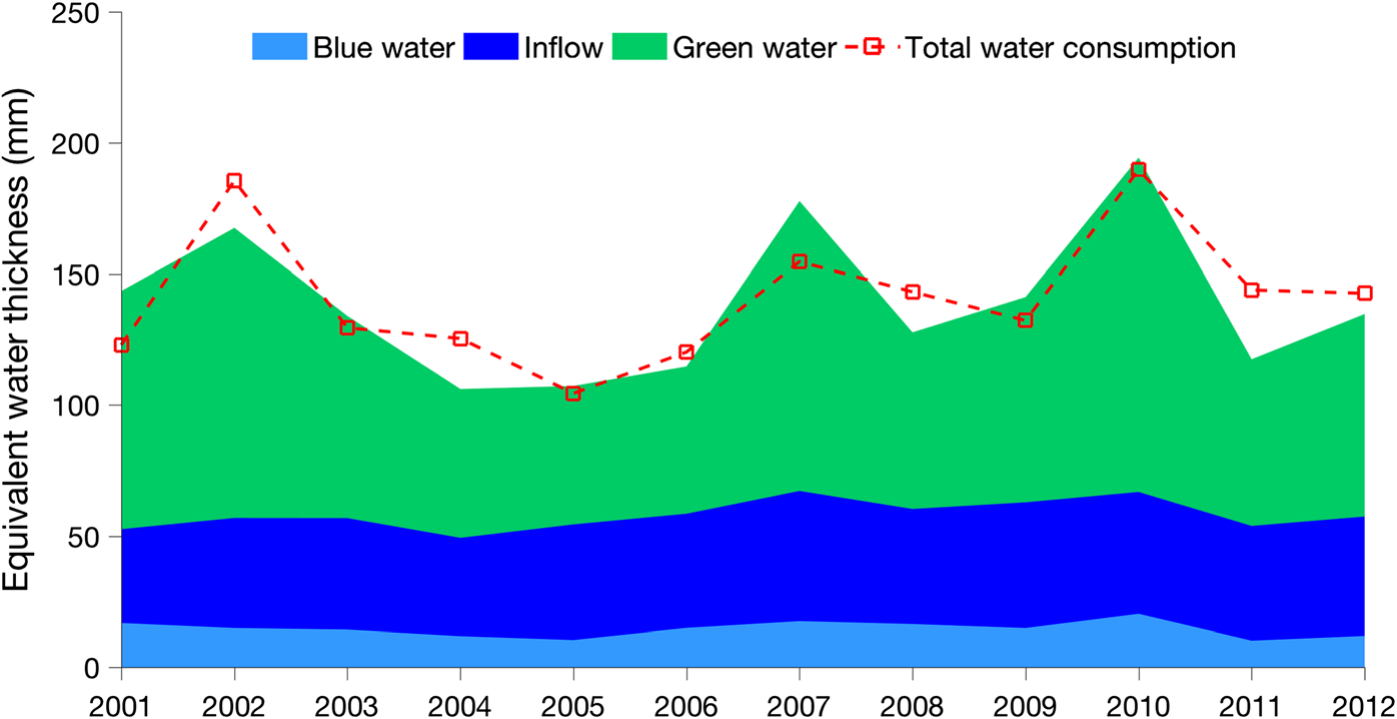
Temporal distribution of the water resources and water consumption. The data from all the pixels for the entire domain were summed and then converted into equivalent water thickness for the sake of convenience for comparison. The light blue indicates the blue water from precipitation, and the green represents the green water from precipitation, while the dark blue represents the blue water from upstream, i.e. the inflow of the domain. The red dashed line shows the water consumption through evapotranspiration

The temporal variability of the total water availability and total water consumption was also analysed and is shown in Fig. 5. In this figure, the different colours of blue indicate blue waters from different sources. The dark blue indicates the blue water from upstream, i.e. inflow, while the light blue represents the blue water from precipitation. Both the total water resources (including the green water, blue water and the inflow from upstream) and the total water consumption (evapotranspiration) vary over time, while the green water is still the main water resource for each year with an increased bandwidth in the figure. The sum of green and blue water represents the total precipitation. On visual inspection, a clear pattern emerges that shows that the annual variability of green water depends very much on the temporal distribution of precipitation for this region. This is in line with the findings of other studies in this region (Zang et al. 2012). Moreover, green water resources have greater temporal variability from precipitation (coefficient of variation: 30.2%) than blue water resources (coefficient of variation: 20.8%). A statistical analysis also shows a steady annual pattern for inflow, with the coefficient of variation at only 9%. The water consumption follows an annual pattern similar to that of the total water resources. This similarity is because water availability is the main constraint for consumption in this region (Elliott et al. 2014). However, there is still inequality between the total water resources and the total water consumption, and this imbalance reflects the total water storage change in the entire domain.

### Explicit green and blue water analysis for different ecosystems

**Fig. 6:**
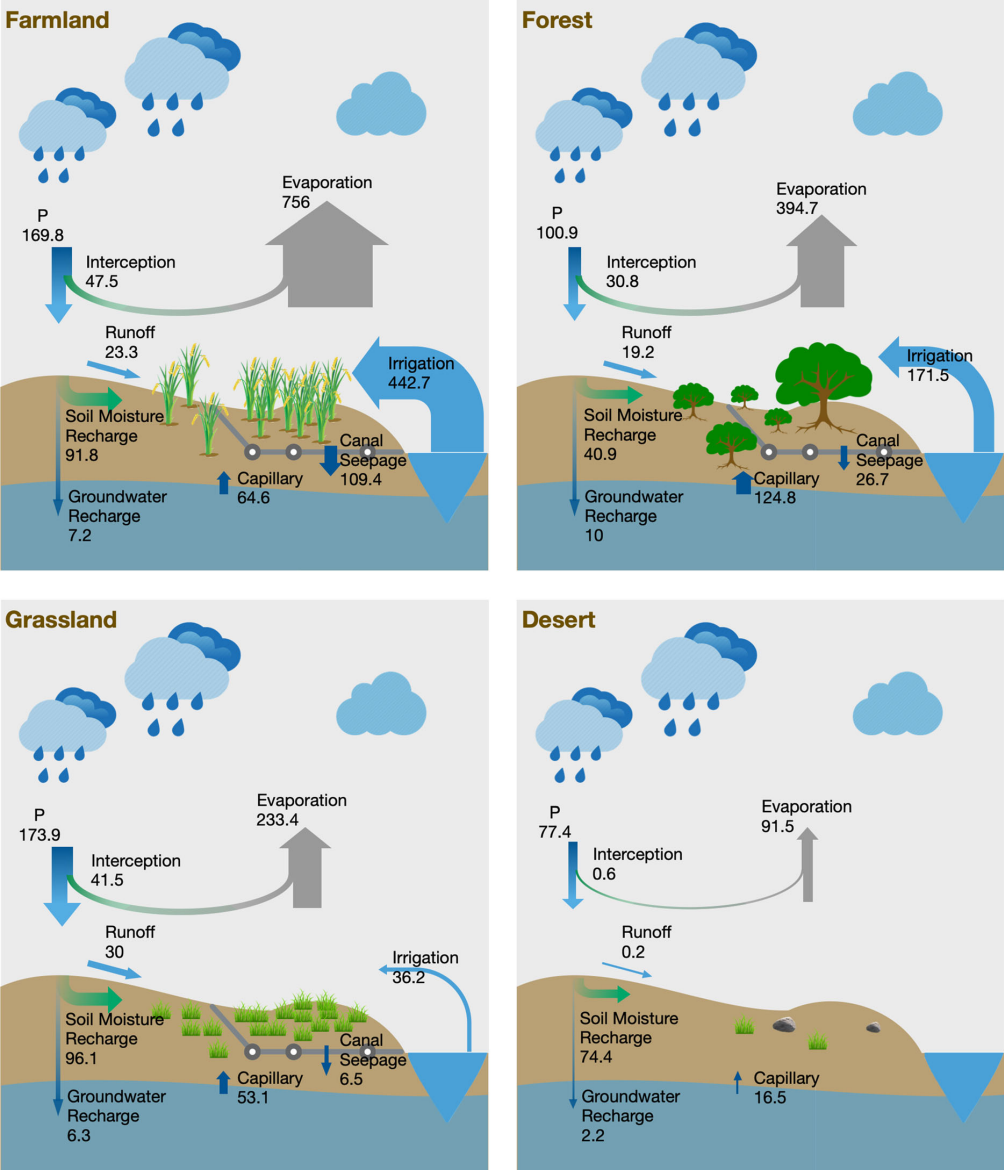
Explicit green and blue water assessment for different ecosystems on an annual scale. The data are summed from all the pixels from a certain ecosystem and then converted into equivalent water thickness. The unit is mm/year for all four plots. Blue arrows indicate blue water flows, and green arrows indicate green water flows. Arrows with a gradient transition from blue to green represent the transformation of blue water to green water. The size of the arrow reflects the magnitude of water flows

Different ecosystems have different hydrological processing mechanisms; thus, the philosophy of water use for different ecosystems might also be different (Gao et al. 2017; Savenije and Hrachowitz 2017). In addition, the spatial and temporal heterogeneity shown in previous analyses will also affect water use by different ecosystems. Therefore, it is necessary to investigate the blue and green water regimes in different ecosystems. In this section, the green and blue water assessment is performed for different ecosystems. Here, four major ecosystems, i.e. farmland, forest, grassland and desert, are included, and the results of the explicit investigations are shown in Fig. 6. Different from the gross water flow chart investigation (Fig. 3) that reflects the total amount of different flows, this figure shows the water flows at depth (unit: mm/year). This allows us to see the intensity of water fluxes and their exchanges and provides a clear impression by representing the amount with corresponding arrow size.

Regarding the water resources, our investigation shows that the grassland ecosystem receives the highest amount of precipitation per unit area (173.9 mm/year), which is very close to the precipitation received by the farmland ecosystem (169.8 mm/year). The annual precipitation for the forest ecosystem is 100.9 mm/year, and for the desert ecosystem, the amount is only 77.4 mm/year on average. Precipitation partitioning is the critical process for green and blue water formation, and the partitioning ratio is different for the different ecosystems because land cover has a significant influence on run-off generation (Hernandez et al. 2000; Sriwongsitanon and Taesombat 2011). The GWCs for the farmland, forest, grassland and desert are 0.82, 0.71, 0.79 and 0.97, respectively. This means that the forest ecosystem has the highest run-off coefficient and the desert ecosystem has the lowest run-off coefficient where precipitation is mainly stored in the soil rather than running off. Even though interception is accounted for as green water, it is still separate from the total evaporation in our investigation because it is directly evaporated from leaves back into the atmosphere and does not infiltrate the soil. The interception values for farmland, forest, grassland and desert are 47.5 mm/year, 30.8 mm/year, 41.5 mm/year and 0.6 mm/year, respectively. Irrigation was not intercepted because flood and furrow irrigation were still the main irrigation modes in the HRB during our research period (Zhou et al. 2015a, b). There is also interception in the desert area since some regions considered in our model are covered by desert vegetation. The blue water consists of surface run-off, subsurface run-off and groundwater recharge, and the percentage of each component is different due to the different mechanisms of hydrologic cycling in ecosystems (Savenije and Hrachowitz 2017). To provide a clear representation of the movement of green and blue water without making the plots unnecessarily complex, some fluxes are purposefully combined, i.e. the surface run-off and subsurface run-off are combined as run-off in Fig. 6. Even though the surface run-off and subsurface run-off have different hydrological process mechanisms, they play similar roles in the green and blue water flow chart from the perspective of water resource assessment.

The water consumption for these four ecosystems is quite different, varying from 91.5 mm/year in the desert ecosystems to 756 mm/year in the farmland ecosystems. Although the desert consumes most of the water resources in this region (see Fig. 3), the consumption per unit area is low (91.5 mm/year). The farmland ecosystem has the highest water consumption per unit area, at 756 mm/year, which is partly due to the large amount of irrigation. Our analysis shows that except for the desert, the other ecosystems received additional water resources through different magnitudes of irrigation because irrigation also happens in the forest and grassland ecosystems (see "Study area" section). The farmland was irrigated at a rate of 442.7 mm/year per unit, which is more than three times the amount of green water stored in the soil. This follows the general pattern in arid regions, where the water for agriculture is limited; thus, agriculture requires more water through irrigation (Al-Zu’bi 2007). The forest also received 171.5 mm/year of irrigated water, and the irrigation rate for the grassland was only 36.2 mm/year. Even though the unit-averaged irrigation rate for the grassland is lower than that for the forest, the received amount of irrigation for the grassland is larger due to the larger area of the grassland compared to the forest (see "Study area" section). Regarding the volume of irrigated water, the grassland received 7.4% of the total irrigation (0.26 billion m^3^/year), the forest received 6.6% of the total irrigation (0.24 billion m^3^/year), and the farmland received 86% of the irrigation (3.13 billion m^3^/year). Moreover, a large amount of water from the irrigation canal seeps out of the canal, i.e. canal seepage. This water is also an important source for ecosystems, as the water seepage recharges the soil and can be used by plants. This kind of soil moisture recharge, including irrigation, is different from the soil moisture recharge from precipitation, as the water is reallocated from the stream to the soil and will be accounted for by blue water consumption instead of green water consumption (Scott et al. 2000). The amount of canal seepage very much depends on the magnitude of irrigation, and it also reflects the water use efficiency of irrigation (Wang et al. 2015). In addition to human-induced blue water redirection, irrigation and canal seepage, there is also physically induced soil moisture recharge, e.g. capillary uptake, which draws groundwater and provides a considerable amount of water for ecosystems (Zhang 2016). The capillary uptake for the forest is 124.8 mm/year, which is even higher than the soil water recharge from precipitation. The capillary uptake for the farmland, grassland and desert is much less, at 64.6 mm/year, 53.1 mm/year and 16.5 mm/year, respectively. This difference is because the roots of plants in the forest can reach deep soil, and transpiration can cause a higher moisture deficit pressure than that in the other ecosystems (Zhu et al. 2009).

### Water consumption dynamics between humans and nature

The HRB is located in an arid region, where the water resources are limited for both humans and natural ecosystems. However, agriculture, which requires a large amount of water, is important for the local economy in the HRB (Cheng et al. 2014). The farmland ecosystem, i.e. the human ecosystem, requires a considerable amount of blue water, as this ecosystem consumes water extensively through irrigation (see "Domain-averaged green and blue water flow chart" and "Explicit green and blue water analysis for different ecosystems" sections). However, the use of water by humans will undoubtedly reduce the water available to natural ecosystems, such as forest, grassland ecosystems and desert ecosystems. To balance the water consumption between humans and nature, a holistic understanding of water consumption dynamics for both humans and nature is necessary.

**Fig. 7:**
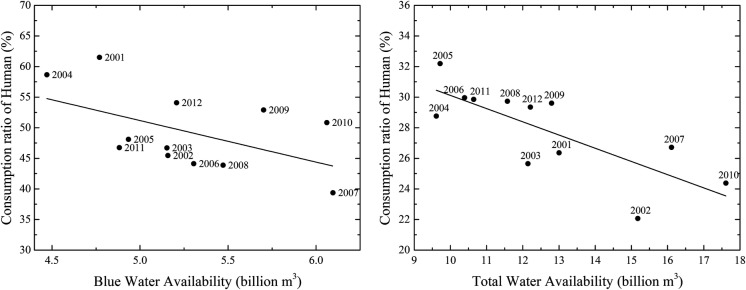
The relationship between the consumption ratio for humans and water availability. The left plot shows the results based on blue water availability and the blue water consumption ratio, while the right plot shows the results based on the total water availability and the total water consumption ratio. The black dots represent the consumption ratio for humans in different years. The solid line represents the linear regression of the data. All the results are calculated on an annual scale based on the data from 2001 to 2010

In this section, the relationship between human water consumption and natural water use is investigated, and the results are shown in Fig. 7. The water consumption ratios are calculated for both humans and nature, while only the results for humans are shown in the plots, as the consumption ratio of nature equals one minus the consumption ratio of humans. In this work, the water consumption by the forest, grassland and desert are considered natural water use, and the water consumption for farmland is considered human water use. Therefore, the consumption ratio of humans refers to the fraction of water use by the human ecosystem, i.e. the water consumption ratio for farmland. Here, we consider only agricultural water use as human water use since the sum of domestic and industrial water use is less than 5% of the total human water use in the entire HRB (Cheng et al. 2014; Li et al. 2015; Wang et al. 2009). Moreover, domestic and industrial water use is irrelevant to green water and blue water interactions since almost all of the return water from domestic and industrial water abstraction flows back into the river channel through pipes, causing no exchange between the green and blue water (Vanham et al. 2018). Indeed, neglecting domestic and industrial water use will influence the calculation of the total water consumption; however, the results will be only slightly affected due to the low volume of water, and we are more interested in the ecosystem water consumption from the perspective of hydrology, which can reflect the interlinkages between green and blue water.

Figure 7 shows that both the BWCR and total water consumption ratio for humans vary in different years. The human consumption ratio of blue water ranges from 39.4% in 2007 to 61.5% in 2001, while the human consumption ratio of total water ranges from 22.1% in 2002 to 32.2% in 2005. A linear regression (Fig. 7: solid line in plots) shows the linear relationship between water availability and human water consumption. The human consumption ratio of blue water is relatively higher than that of total water, and both the BWCR and total water consumption ratio for humans show a clear decreasing trend with increasing corresponding water availability. This trend exists because blue water use can be controlled by humans through water allocation. Water resources for humans are a higher priority than those for nature, as the water consumed by human ecosystems, i.e. farmland, benefits the local economy. In dry years, e.g. 2004, nearly 60% of the blue water resources are used by the human ecosystem. Since the green water resources for crops are extremely insufficient, additional water resources are required. In wet years, e.g. 2007, water stress is low and few blue water resources might be sufficient to meet the water demands of crops. In this case, more blue water resources will be available to the natural ecosystem, and the BWCR for humans will be low (39.4%).

Our results also reveal that natural ecosystems may face an increased risk of water consumption (especially blue water consumption), as the human consumption ratio increases when the water availability decreases. In other words, increasing competition for water between humans and nature will result in restrictions on natural water use. To investigate the potential risk that ecosystems may face under changing environments under water competition, an analysis of the reliance of water consumption on blue water has been performed, and the details are described in the next section.

### Identification of hotspots at potential risk of water competition

Even though green water is the main water resource in the HRB region, the importance of blue water is also crucial (see "Domain-averaged green and blue water flow chart" section). However, the high reliance on blue water may result in a potential risk of increased water competition since this reliance may transgress regional constraints on freshwater use as a result of the overconsumption of blue water; consequently, ecosystems might experience increased conflicts and tensions over water resources (Johansson et al. 2016).

**Fig. 8:**
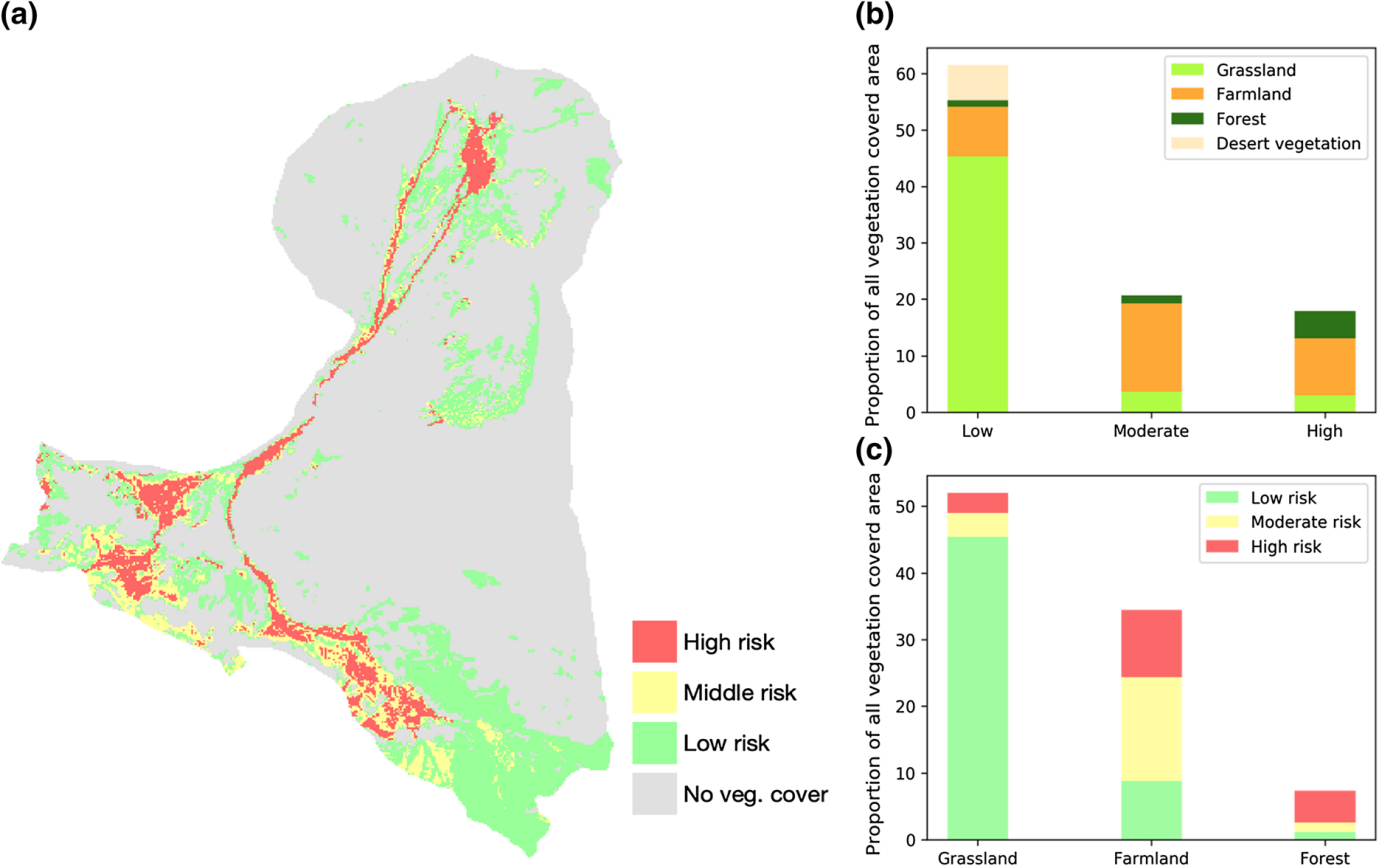
BWCR-based analysis of potential risk of increased water competition including **a** the risk map illustrating the identified hotspots, **b** land use (ecosystems) components of the risk areas and percentages of different risk levels, **c** risk information for different ecosystems as a percentage

To assess the potential risks that ecosystems may face, an index called the BWCR (see "Green water coefficient and Blue Water Demand Index" section) is applied. The BWCR index represents the blue water consumption ratio of a certain region or pixel. A high BWCR value indicates a high dependence on blue water resources, thus reflecting a high potential risk of increased freshwater competition. Here, we established two BWCR thresholds to identify the high-risk, moderate-risk and low-risk areas, which also reflects the sensitivity of the region to freshwater availability. BWCR indices are calculated for all areas with vegetation cover in the research domain, and the results are illustrated in Fig. 8.

Figure 8a shows the risk map of potential increased competition over water resources, and coloured pixels indicate different levels of risk. The BWCR-identified areas with low risk are mainly distributed in the area where the annual precipitation is relatively high, e.g. in the south-east of the domain (see Fig. 4). In this region, precipitation produces more green water resources than in other regions. Thus, the dependence on blue water, i.e. freshwater, in this region is relatively low. In the northern part of the domain, there are some regions with a low potential risk of water competition, even though the precipitation amount is low mainly because these regions are covered by grass or desert vegetation, which requires less water to survive. The BWCR-identified areas with moderate or high risk are mostly present in the irrigated area. This is inconsistent with the definition of the BWCR, as the BWCR indicates the water consumption dependency on blue water. Irrigation redirects a large amount of freshwater to these regions. Interestingly, we found that areas along the river also have a high potential risk of increased water competition. The main reason for such a high potential risk could be that plants can take advantage of a relatively high groundwater table in the nearby river area and obtain groundwater through capillary uptake. The risk map (Fig. 8) allows us to visualize the spatial heterogeneity and identify hotspots that may face a high potential risk of increased water competition for water consumption in future. The map, especially the detailed spatial information, provides critical data for sustainable water management.

In addition to spatial hotspot identification, our analyses also show the proportions of different risk levels and different ecosystems (Fig. 8b). For the entire domain, the vegetation-covered area accounts for 29% of the total area of the region, and the following values were calculated based on only the vegetation-covered area. Summing the different risk levels in the specified area shows that 17.9% of the area is at a high risk of increased water competition and relies almost completely on blue water resources. These areas comprise a small percentage of grassland, a large percentage of farmland and a small percentage forest; however, most of the forest is contained within this area. Approximately 20.7% of the area under moderate risk of increased water competition requires more than 50% of water resources from blue water. This area is dominated by farmland and includes a small proportion of forest and grassland. The rest of the area (61.4%), which is dominated by grassland, is considered to have a low risk of water competition in future. Notably, all the vegetation-covered desert areas are with low risk because of the nature of desert plants. There are also some farmland areas categorized as low-risk areas; they are mostly distributed in the south-east of the domain where the precipitation is high. By investigating the risks for different ecosystems (Fig. 8c), we can see that most grassland ecosystems are categorized as having a low risk, and nearly all forest-cover areas are categorized as having a moderate or high risk of increased competition for water use. The risk proportions for farmland are approximately even, meaning approximately 1/3 of the area might be vulnerable to a high risk of water competition.

The identified hotspots with moderate or high risk are very sensitive to freshwater availability, as their ecosystems require more than 50% of the blue water. These areas mainly include farmland (human ecosystem) and forest (natural ecosystem) and should be considered a higher priority for water resource management. As the blue water resources are used by the human ecosystem, which has a higher priority than the natural ecosystem (see "Water consumption dynamics between humans and nature" section), the forest ecosystem could experience increased pressure for blue water resource use. The identification of hotspots combined with the analysis of the dynamics between human and natural water use in the HRB region, especially spatial risk information and separate estimations for different ecosystems, could provide crucial implications for water management in this region. Moreover, in order to make the water management more in practical, an arbitrary threshold value is used to separate high, moderate and low risks at the moment in this study. This could bring uncertainties for general application of this method, as the ecosystems could have different characteristics in different regions. This should be taken into consideration in follow-up researches.

## Conclusion

In this study, we explicitly assessed green and blue water resources by considering the interactions between green and blue water as much as possible in the HRB, the second largest endorheic river basin in China. An integrated hydrological model is applied to simulate complex eco-hydrological processes and provide essential hydrological simulations for this assessment. Major findings of this study include the following:

- Even though the green water resources are the major resources in the research area, an arid river basin, the upstream blue water resources are also crucial to meet the water demand of the ecosystems in this region. The redirection of blue water to soil moisture plays a key role in the complete water cycle in this area, as the water required for evaporation is much higher than the water stored in the root zone from precipitation.
- Both the water availability and water consumption vary in time and space in the research area. Different hydrological process mechanisms in the ecosystems, together with the spatial and temporal heterogeneity of the water supply, form totally different green and blue water regimes in different ecosystems. The farmland ecosystem relies greatly on irrigation, while the forest relies on both irrigation and capillary uptake. Both the grassland and desert ecosystems mainly rely on green water from precipitation, while the desert ecosystem generates almost no run-off.
- The historical relationship between human water use and natural water use indicates that the human ecosystem has a higher priority than the natural ecosystem for blue water resources. The water consumption ratio for humans increases with the decrease in water availability. Natural ecosystems may experience increased pressure when the water competition between humans and nature increases.
- A new index called the BWCR is proposed to identify the hotspots that may face a potential risk of increased water competition for blue water use. Approximately 38.6% of the vegetation-covered areas are categorized as hotspots that may face moderate or high risks of increased conflict and tension over freshwater. These areas are dominated by farmland and forest. The forest is the most vulnerable ecosystem in the HRB. The situation for natural ecosystems could even be worse, as the human ecosystem, i.e. farmland, will have a higher priority for freshwater use.

This study assesses water resources by considering not only blue and green water but also their interconnections in the HRB. By taking advantage of a sophisticated hydrological model, this study helps us to better understand the interactions between green and blue water, especially in different ecosystems. This study also investigates blue and green water from both the water supply and water consumption perspectives. Such a sophisticated research framework allows us to consider all of the important factors in the water resource assessment. The detailed analyses of green and blue water dynamics bring us a step closer to understanding human and natural water use dynamics. A new water assessment index has been developed to identify the hotspots that might face increased conflict and tension over blue water resources. This study highlights the need to consider the interactions between green and blue water for water resources assessments because there is strong exchange between the compartmentalized stores of water (McDonnell 2017), especially in the areas with high human activity, which may redirect a large amount of blue water back into soil. The risk analysis for increased water competition for different ecosystems provides essential information for water management under the changing environment; these data can help maintain the balance between humans and nature and promote sustainable development.

However, in this study, there are a few limitations. First, the current work omitted industrial and domestic water uses due to a lack of data. Even though these uses did not influence the interactions between green and blue water much, the calculation of water consumption for humans is slightly affected, thus causing a small impact on the results of water consumption dynamics between humans and nature. Second, the results are simulated with one model. Although the model has been calibrated and validated in several previous studies in the same region (Li et al. 2017, 2018; Tian et al. 2015a, b), simulations may be constrained by the fundamental assumptions and approaches used in this model.
